# Navigating the Unknown: Scleroderma Renal Crisis and a Fatal Cardiac Complication in a Young Patient With MCTD With Coexisting Anti‐U3 RNP and Anti‐RNA Polymerase III Antibodies

**DOI:** 10.1155/crrh/5033806

**Published:** 2026-09-07

**Authors:** Tiago Reyes-Castro, Amir Nabizadeh, Rawad Nasr, Linh Ngo

**Affiliations:** ^1^ Department of Medicine, University of Minnesota, Minneapolis, Minnesota, USA, umn.edu; ^2^ Department of Medicine, Division of Rheumatology, Hennepin County Medical Center, Minneapolis, Minnesota, USA, hcmc.org

## Abstract

We report the case of a 30‐year‐old Somali female with mixed connective tissue disease (MCTD) who developed dermatomyositis and both scleroderma renal crisis (SRC) and fatal cardiac complications. Notably, she tested positive for both anti‐U3 RNP and anti‐RNA polymerase III antibodies which is a rare serologic combination. After initial treatment with corticosteroids for dermatomyositis features, she experienced rapid clinical deterioration marked by malignant hypertension and thrombotic microangiopathy and was found to have SRC and diffuse myocardial fibrosis. Despite treatment with captopril and hemodialysis, she ultimately died of cardiac arrest. This case highlights the complexity and risks in managing patients with overlapping autoantibodies and underscores the need for close monitoring and cautious use of steroids in such high‐risk individuals.

## 1. Introduction

Scleroderma renal crisis (SRC) is a rare but life‐threatening complication of systemic sclerosis (SSc) and mixed connective tissue disease (MCTD), often precipitated by corticosteroid use [1]. It is characterized by acute kidney injury, malignant hypertension, and thrombotic microangiopathy (TMA), with significant morbidity and mortality despite advances in treatment [1, 2]. The presence of specific autoantibodies, such as anti‐RNA polymerase III and anti‐U3 ribonucleoprotein (RNP), has been associated with distinct clinical phenotypes, including early‐onset disease, worse prognosis, and an increased risk of SRC [3]. However, the coexistence of these two antibodies in a single patient is exceedingly rare, with limited reports in the literature.

We present a young Somali female with MCTD who developed SRC and fatal cardiac complications. This case underscores the need for early recognition and tailored treatment approaches in high‐risk patients.

## 2. Case Presentation

A 30‐year‐old Somali female with a history of vitamin D deficiency, currently breastfeeding, presented with 2 months of progressive fatigue and proximal muscle weakness, predominantly affecting her lower extremities. She also reported a hypopigmented rash on her fingernails, periorbital region, and right knee, along with bilateral hand and foot pain, stiffness, and decreased grip strength. She denied any dry eyes, dry mouth, Raynaud’s phenomenon, or dysphagia. She denied prior autoimmune conditions, medication use, tobacco, alcohol, or illicit drug use. Family history was negative for autoimmune diseases.

On examination, vital signs were stable, with a baseline blood pressure of 111/78 mmHg. Skin findings included hypopigmentation over the eyelids and left knee with periungual thickening. There was no nail pitting, alopecia, or mechanic’s hands. Musculoskeletal exam revealed proximal muscle weakness, with discomfort on movement but no joint swelling. Neurologic exam was intact aside from reduced muscle effort. Laboratory studies showed elevated creatine kinase of 596 IU/L, elevated inflammatory markers, including an erythrocyte sedimentation rate of 46 mm/hr, CRP of 12 mg/L, and lactate dehydrogenase of 403 U/L. Antinuclear antibody was positive at a titer of > 1:2560 with a homogeneous pattern, positive anti‐U3 RNP, and positive anti‐RNA polymerase III. Baseline renal function was normal with a creatinine of 0.8 mg/dL and a urine protein‐to‐creatinine ratio (UPCR) of 0.14 mg/mg. Urinalysis showed positive blood without red blood cells and no significant proteinuria. Myositis Panel showed + Smith/RNP ab, IgG; +Fibrillarin (U3 RNP) Ab, IgG. MRI of the bilateral lower extremities demonstrated fluid signal along the myofascial boundaries of the right vastus intermedius, gluteus minimus, and adductor magnus, consistent with inflammatory myositis. Anti‐Smith antibody (ab), SSA and SSB ab, anti‐CCP and rheumatoid factor (RF) ab, ANCA and MPO/PR3 ab, and TB quant and hepatitis B and C screenings were negative.

**FIGURE 1:**
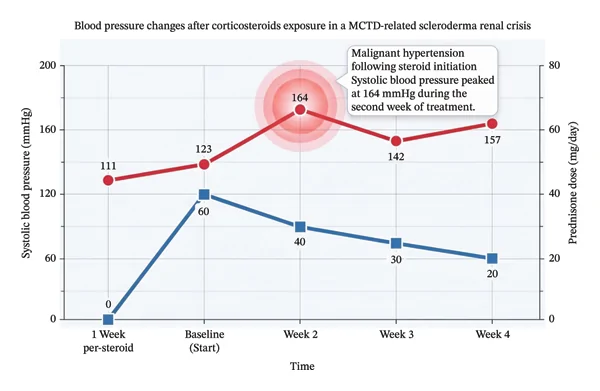
Temporal relationship between systolic blood pressure and prednisone dose following corticosteroid initiation.

**FIGURE 2:**
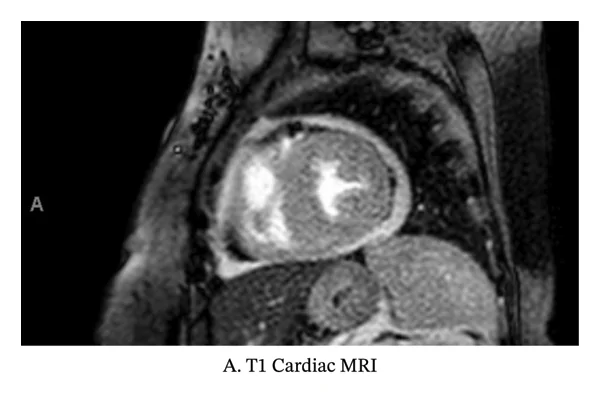
Cardiac MRI showing elevated native T1 suggestive of diffuse myocardial fibrosis.

She was diagnosed with MCTD with dermatomyositis features based on the characteristic clinical presentation, elevated muscle enzymes, and MRI findings. She was started on high‐dose prednisone (60 mg daily with taper), leading to partial improvement in skin findings but persistent weakness. However, 2 weeks later, an increase in blood pressure (Figure 1), urinalysis demonstrating blood and protein with > 20 red blood cells per high‐power field, and an increase in the UPCR to 1.16 were noted during follow‐up, warranting rapid tapering of prednisone and consideration of intravenous immunoglobulin therapy (IVIG). Shortly after IVIG initiation, she developed shortness of breath, palpitations, and right‐sided chest pain, requiring hospitalization. Vital signs revealed hypertension (164/109 mmHg) and tachycardia (124 bpm). Workup showed findings suggestive of microangiopathic hemolytic anemia, including thrombocytopenia, low haptoglobin, elevated LDH, and rare red blood cell fragments on peripheral smear. Additional findings included troponin elevation (168 ng/L), BNP 5000 pg/mL, and proteinuria (UPCR 1.78). Electrocardiograms demonstrated sinus tachycardia with left posterior fascicular block and nonspecific ST‐T wave abnormalities. The echocardiogram showed severely increased left ventricular (LV) hypertrophy, small pericardial effusion, normal LV ejection fraction, normal right ventricular size and function, and no evidence of pulmonary hypertension. Cardiac MRI showed bilateral pleural effusions, a small pericardial effusion, LV hypertrophy, and diffuse myocardial fibrosis (Figure 2). There was no evidence of late gadolinium enhancement or active myocardial inflammation; however, elevated native T1 and extracellular volume were consistent with diffuse myocardial fibrosis. Coronary angiography was not performed because the clinical presentation favored systemic inflammatory cardiac involvement rather than acute coronary syndrome. Additionally, fundoscopic examination was not documented during the hospitalization, representing a limitation of this case report.

**FIGURE 3:**
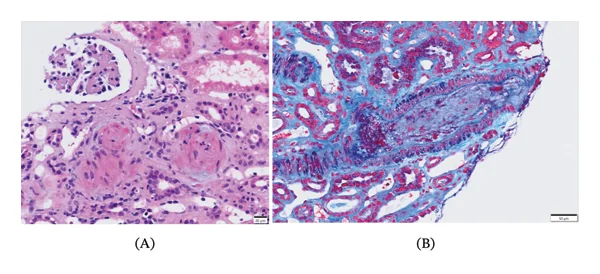
Kidney biopsy showing thrombotic microangiopathy with interstitial fibrosis and tubular atrophy, consistent with scleroderma renal crisis. (A) TMA with schistocytes, ischemic glom, H&E and (B) TMA with schistocytes, trichrome stain.

Given concerns for SRC versus hypertensive emergency or lupus nephritis, nephrology and cardiology teams were consulted. A kidney biopsy revealed TMA with interstitial fibrosis and tubular atrophy, confirming SRC (Figure 3). She was started on captopril, leading to transient blood pressure improvement. However, she subsequently developed biopsy‐related bleeding, worsening kidney function (creatinine rising to 3.62 mg/dL), persistent hypotension, anemia, and hyperkalemia, requiring discontinuation of angiotensin‐converting enzyme (ACE) inhibitors. Hemodialysis was initiated, and mycophenolate mofetil was started for suspected cardiac involvement.

On hospital Day 20, she developed acute respiratory distress, hypotension, and hypoxia, leading to cardiac arrest and death despite resuscitative efforts.

## 3. Discussion

The coexistence of anti‐U3 RNP and anti‐RNA polymerase III antibodies in SSc spectrum disorders is exceptionally rare [4]. Anti‐U3 RNP antibodies are more frequently observed in African American patients and are associated with skeletal muscle involvement, pulmonary arterial hypertension, and worse survival. In contrast, anti‐RNA polymerase III is strongly associated with rapidly progressive skin thickening, malignancy risk, and, importantly, SRC [3]. The presence of both antibodies in this patient creates a unique and high‐risk clinical phenotype with overlapping features, making disease trajectory difficult to predict.

This case highlights the well‐recognized but clinically challenging association between corticosteroid use and SRC. High‐dose corticosteroids are a known precipitating factor for SRC, particularly in patients with anti‐RNA polymerase III antibodies [1].

Given this risk, alternative immunosuppressive strategies should be considered. Steroid‐sparing approaches, including intravenous immunoglobulin (IVIG), mycophenolate mofetil, and rituximab, may offer safer options in high‐risk patients, although data remain limited [4].

An additional important aspect of this case is the presence of significant cardiac involvement. While IVIG has been associated with volume overload and cardiac complications, the patient’s presentation, including malignant hypertension, TMA, and cardiac MRI findings of diffuse myocardial fibrosis supports SRC‐related cardiac involvement rather than treatment‐related toxicity. Cardiac MRI did not demonstrate late gadolinium enhancement or features consistent with active myocarditis, but elevated native T1 and extracellular volume were suggestive of diffuse myocardial fibrosis.

Management of SRC relies on prompt initiation of ACE inhibitors, which remain the cornerstone of therapy [1, 4]. Despite treatment, outcomes remain poor, particularly in severe cases with TMA, with mortality approaching 20% at 6 months [4].

In refractory cases, particularly those with SRC‐associated TMA, additional therapies such as plasma exchange and complement inhibition with eculizumab have been increasingly reported. Eculizumab has demonstrated clinical improvement in the majority of reported cases and is supported by growing evidence implicating complement activation in SRC pathogenesis [5, 6]. Similarly, plasma exchange may be beneficial in selected patients with microangiopathy, although evidence remains limited to observational studies [7].

In summary, this case illustrates a rare and high‐risk presentation of MCTD with coexisting anti‐U3 RNP and anti‐RNA polymerase III antibodies, complicated by SRC and fatal cardiac involvement. It underscores the importance of cautious corticosteroid use, early recognition of SRC, and consideration of emerging therapies in refractory disease.

## Author Contributions

Tiago Reyes‐Castro conceptualized the report, performed the literature review, drafted the manuscript, prepared the figure, and led the revision process. Amir Nabizadeh contributed to data collection and literature review and critically reviewed the manuscript. Dr. Linh Ngo and Dr. Rawad Nasr provided clinical expertise, critically reviewed the manuscript, and contributed to the revision and final editing of the manuscript.

## Funding

This work received no specific grant from any funding agency in the public, commercial, or not‐for‐profit sectors.

## Disclosure

All authors reviewed and approved the final version of the manuscript.

## Ethics Statement

Ethical approval was not required for this single‐patient case report in accordance with institutional policy.

## Consent

Informed consent for publication of this case report and the accompanying images was obtained from the patient’s next of kin.

## Conflicts of Interest

The authors declare no conflicts of interest.

## Data Availability

The data supporting the findings of this case report are available from the corresponding author upon reasonable request. Additional patient information has not been made publicly available to protect patient privacy and confidentiality.
